# Increased SIX‐1 expression promotes breast cancer metastasis by regulating lncATB‐miR‐200s‐ZEB1 axis

**DOI:** 10.1111/jcmm.15185

**Published:** 2020-03-30

**Authors:** Lizhe Zhu, Siyuan Jiang, Shibo Yu, Xiaoxu Liu, Shengyu Pu, Peiling Xie, Heyan Chen, Xiaoqin Liao, Ke Wang, Bin Wang

**Affiliations:** ^1^ Department of Breast Surgery the First Affiliated Hospital of Xi’an Jiaotong University Xi'an China

**Keywords:** BC, EMT, lncATB, miR‐200s, SIX‐1

## Abstract

Patients with advanced breast cancer (BC) showed a higher incidence of regional and distant metastases. Sine oculis homeobox homolog 1 (SIX‐1) has been confirmed to be a key tumorigenic and metastatic regulator in BC progression. Yet, molecular mechanisms behind SIX‐1‐induced BC metastases remain largely unknown. Here we found that SIX‐1 was frequently up‐regulated in BC and correlated with poor outcomes when tested in human BC tissue microarray. Then, we manipulated the expression of SIX‐1 by via shRNA‐mediated knockdown and lentivirus‐mediated overexpression. Transwell assay in vitro and lung metastases model of nude mice in vivo* showed that* SIX‐1 promoted BC cell invasion and migration in vitro, and facilitated metastases in vivo*.* Mechanistically, SIX‐1 could promote the transcription of lncATB, which exerts critical pro‐metastatic role in BC by directly binding to the miR‐200 family, especially for miR‐200c, to induce EMT and promote metastases. In conclusion, SIX‐1 exerts its pro‐metastatic role in BC through lncATB/miR‐200s axis of EMT signalling pathway and could act as an important diagnostic marker as well as a significant therapeutic target for clinically advanced BC.

## 1 BACKGROUND

Breast cancer (BC) remains one of the most heterogeneous and aggressive malignancies worldwide, despite great improvements have been made in the treatment of BC.^1^, ^2^ Metastases are the primary cause for high rate of tumour related death of advanced BC.^3^, ^4^ Therefore, identifying and elucidating the key mechanisms that contribute to BC metastases is urgently needed. Epithelial‐mesenchymal transition (EMT), which is characterized by losing of epithelial differentiation and acquiring of mesenchymal‐like cellular capacity, is well recognized to be the major cause of tumour metastases.^5^, ^6^, ^7^ However, the molecular basis behind EMT‐mediated BC metastases remains largely elusive. The sine oculis homeobox homolog 1 (SIX‐1) homeoprotein, which is frequently overexpressed in advanced malignancies, has been confirmed to be a key tumorigenic and metastatic regulator in BC progression.^8^ SIX‐1 was primarily identified as overexpressed in BC cells and contributed to gene amplification and chromosomal instability in BC.^9^, ^10^, ^11^ Mechanistic insights come from the previously studies further confirmed that SIX‐1 served as a metastatic regulator that associated with transforming growth factor β (TGF‐β)‐induced EMT in BC.^12^, ^13^ However, the exact mechanism of SIX‐1 promoting the invasion and metastasis of BC is still largely unclear.

Emerging evidences suggested that dysregulation of microRNAs (miRNAs) plays a key role in tumorigenic and metastatic by controlling downstream genes.^14^, ^15^, ^16^ Among which, miR‐200 family has been recognized to be critical in the process of cancer metastasis.^17^ The miR‐200 family (miR‐200s) possess five members organized as two clusters, miRs‐200b/a/429 and miRs‐200c/141.^18^ Previous studies demonstrated that miR‐200 family members could inhibit EMT by directly targeting zinc‐finger E‐box‐binding homeobox factor 1 (ZEB1) and zinc‐finger E‐box‐binding homeobox factor 2 (ZEB2). In turn, ZEB1 and ZEB2 could suppress the miR‐200s promoter activity by binding to the E‐box elements, making a feedback loop with miR‐200s to promote EMT process.^19^, ^20^, ^21^ Recent reports proved that several transcription factors and long non‐coding RNAs (lncRNAs) could control miR‐200s expression in metastases.^22^, ^23^, ^24^ However, the specific transcription factors or lncRNAs that could control miR‐200s expression and regulating EMT to promote metastasis are not well studied.

In this study, we found that SIX‐1 was frequently up‐regulated in BC and correlated with poor outcomes. In addition, SIX‐1 promoted BC cell invasion and migration in vitro, and facilitated metastases in vivo. Mechanistically, SIX‐1 could indirectly promote the transcription of lncATB, which exerts critical pro‐metastatic role in BC by directly binding to the miR‐200 family, especially for miR‐200c, to induce EMT and promote metastases. Furthermore, SIX‐1 may act as an important diagnostic marker as well as a significant therapeutic target for clinically advanced BC.

## 2 MATERIALS AND METHODS

### 2.1 Human tissues collection and treatment

Tissue microarray containing 45 pairs of BC tissues and paired non‐tumour breast tissues were purchased from Outdo Biotech Co. Ltd. A total of 183 of BC tissues and 20 non‐tumour breast tissues were randomly obtained with informed consent from surgery patients of BC (First Affiliated Hospital of Xi'an Jiaotong University, Xi'an, Shaanxi, China). Patients enrolled for this study were not received any chemotherapy, radiotherapy or targeted therapy. The tissues collected were snap‐frozen in liquid nitrogen immediately for further RNA and protein extraction. All the human studies were performed according to ethical consent granted from the Clinical Research Ethics Committee of the Xi'an Jiaotong University.

### 2.2 Cell culture and cell transfection

All the BC cell lines, including MCF‐7, MDA‐MB‐231, MDA‐MB‐361, MDA‐MB‐435, MDA‐MB‐468, ZR‐75‐1 and HS‐578T, were ordered from Cell Bank of Chinese Academy of Sciences (Shanghai, China) and were cultured under normal conditions at 37°C with 5% CO_2_. BC cells were cultured in DMEM medium (Gibco) with 10% foetal bovine serum (Gibco, USA) and 1% antibiotics (Gibco). Cells were tested for mycoplasma contamination using mycoplasma detection PCR and were found to be negative for mycoplasma contamination. For cell transfection, miR‐200a, miR‐200b, miR‐200c, miR‐141 and miR‐429 mimics, ZEB1 siRNA and their corresponding negative controls were synthesized by RiboBio. MCF‐7 or MDA‐MB‐231 BC cells were seeded into 6‐well plates (1 × 10^6^ cells/well) and transfected with miR‐200a, miR‐200b, miR‐200c, miR‐141 and miR‐429 mimics (50 nmol/L), siRNA (100 nmol/L) or the corresponding negative control. SIX‐1 overexpression and knockdown lentiviral vectors and their corresponding control vectors were constructed by GenePharma. All the transfection procedures were conducted following the manufacturer's protocol.

### 2.3 RNA extraction and quantitative real‐time RT‐PCR

Tissues and cells were collected, and Trizol (Invitrogen) was used for total RNA isolation according to the manufacturer's instructions. For messenger RNA (mRNA) expression assay, complementary DNA (cDNA) was obtained from 1.0 μg of total RNA using the PrimeScriptTM RT reagent Kit (Takara Biotechnology, Dalian, China). Real‐time PCR was performed in triplicate with the diluted cDNAs by using iTaq Universal SYBR green PCR system (Takara Biotechnology). Quantitative data of mRNA expression were calculated by using β‐actin as an internal control. For microRNA (miRNA) expression assay, real‐time PCR was performed in triplicate, and quantitative data of miRNA expression were calculated using U6 as an internal control.

### 2.4 Protein preparation and Western blots analysis

Tissues and cells were lysed in RIPA buffer supplemented with protease inhibitor and phosphatase inhibitor (Thermo Scientific). The protein concentration was evaluated by using BCA Assay Kit (Biyuntian) and then was denatured by using loading buffer (5×) at 100°C for 5 minutes. Proteins with equal amounts (30 μg) were separated by SDS‐PAGE. Primary antibodies used were listed as follows: anti‐E‐cadherin antibody (1:1000, #3195; Cell Signaling Technology); anti‐vimentin antibody (1:1000, #5741; Cell Signaling Technology); anti‐ZEB1 antibody (1:1000, #3396; Cell Signaling Technology); and anti‐SIX‐1 antibody (1:1000, #16960; Cell Signaling Technology). Then, the PVDF membranes were incubated with antimouse or anti‐rabbit HRP‐conjugated secondary antibody (1:1000; ZSGB‐bio). An anti‐β‐actin antibody (1:5000, A5441; Sigma‐Aldrich) was used as an internal control.

### 2.5 Luciferase promoter assays

PmirGLO, pmirGLO‐ATB or pmirGLO‐ATB‐mut (miR‐200), and the plasmid that containing wild‐type ZEB1‐3′‐UTR sequence and a mutant‐type ZEB1‐3′‐UTR sequences were generated by RiboBio (Guangzhou). As shown in Figure 5, the ‘Luc‐ZEB1‐mu’ in each group represented the binding sites of miR‐141, miR‐200a, miR‐200b, miR‐200c and miR‐429 mutated on the wild‐type firefly luciferase reporter constructs, respectively. When cells reached 60% confluence in 24‐well plates, a firefly luciferase reporter gene construct (0.1 μg), miRNA construct (0.4 μg) and a Renilla luciferase construct (0.02 μg) were cotransfected into the cells using X‐tremeGENE HP (Roche, 6366244001). Subsequently, 48 hours after transfection, luciferase activity was measured using a Dual‐Luciferase Reporter Assay System (Promega, E1910) according to the manufacturer's instructions, and the relative luciferase reporter activity was calculated.^22^, ^25^

### 2.6 Wound healing assay

For wound healing assay, cells (5 × 10^5^) were seeded into 6‐well plates until it reaches a confluence of 90%‐95%. Then, the straight wounds were generated by using a 200‐μL plastic sterile tips, and the wound closure was recorded and calculated by using a microscope. Cell migration was analysed using NIH ImageJ software.

#### 2.6.1 Transwell migration and invasion assays

In vitro functional studies of cell migration and invasion were evaluated by transwell migration and invasion assay. Cell migration and invasion capacity were also determined by transwell migration assay by using an 8μm pore size transwell chambers (Corning). Briefly, for migration assay, cells (5 × 10^4^) were seeded in the upper chamber covered with serum‐free medium, while medium supplemented with 20% FBS was added into the lower chamber. After 24 hours of incubation, cell was fixed and stained with 0.1% crystal violet, while cells still reserved on the upper membrane were removed. Cell invasion assay was performed in the same manner but with matrigel covered in the upper chamber previously. The images were taken with inverted microscope (CX41, Olympus) and analysed using NIH ImageJ software.

### 2.7 In vivo experiments

Male nude mice (4‐week‐old, n = 10/group) were purchased from Beijing HFK Biotechnology Co, Ltd. Animal study was approved by the Ethics Committee of the Xi'an Jiaotong University. In vivo metastases were evaluated by injecting cells (3 × 10^6^) through tail vein of the nude mice. After 8 weeks of injection, mice were killed and the lung were removed and fixed. Number and size of metastatic tumour in the lung were calculated by pathologists after haematoxylin and eosin (HE) staining.

### 2.8 Immunohistochemical (IHC) staining

Immunohistochemical analysis for SIX‐1 expression of BC was performed according to standard protocols. Briefly, BC tissue microarray was deparaffinized, rehydrated, washed, the antigen was retrieved, and the endogenous peroxidase activity was blocked. Then, the slide was incubated with anti‐SIX‐1 primary antibody overnight and then with biotinylated secondary antibody for 1 hour. Subsequently, the slide was stained with the diaminobenzidine (DAB) and haematoxylin. The histological scores were evaluated and defined by experienced pathologists in blind as previously described.^26^

### 2.9 Immunofluorescence (IF) staining

Cells were cultured and fixed, and then incubated with primary antibodies, followed by incubation with according secondary antibody. Finally, the cells were stained with 4′,6‐diamidino‐2‐phenylindole (DAPI) (Southern Biotech), and the results were examined by confocal microscope (Olympus).

### 2.10 Statistical analysis

All statistical analyses were performed by using the GraphPad Prism version 5.0 (GraphPad Software, CA) and SPSS 20.0 software (SPSS Inc). Student's *t* test, Pearson chi‐square test, Pearson correlation analysis, Kaplan‐Meier analysis and log‐rank test were used for comparisons as indicated. A *P* value < .05 was considered statistically significant.

## 3 RESULTS

### 3.1 SIX‐1 was frequently up‐regulated in BC, and correlated with poorly outcomes

**Figure 1:**
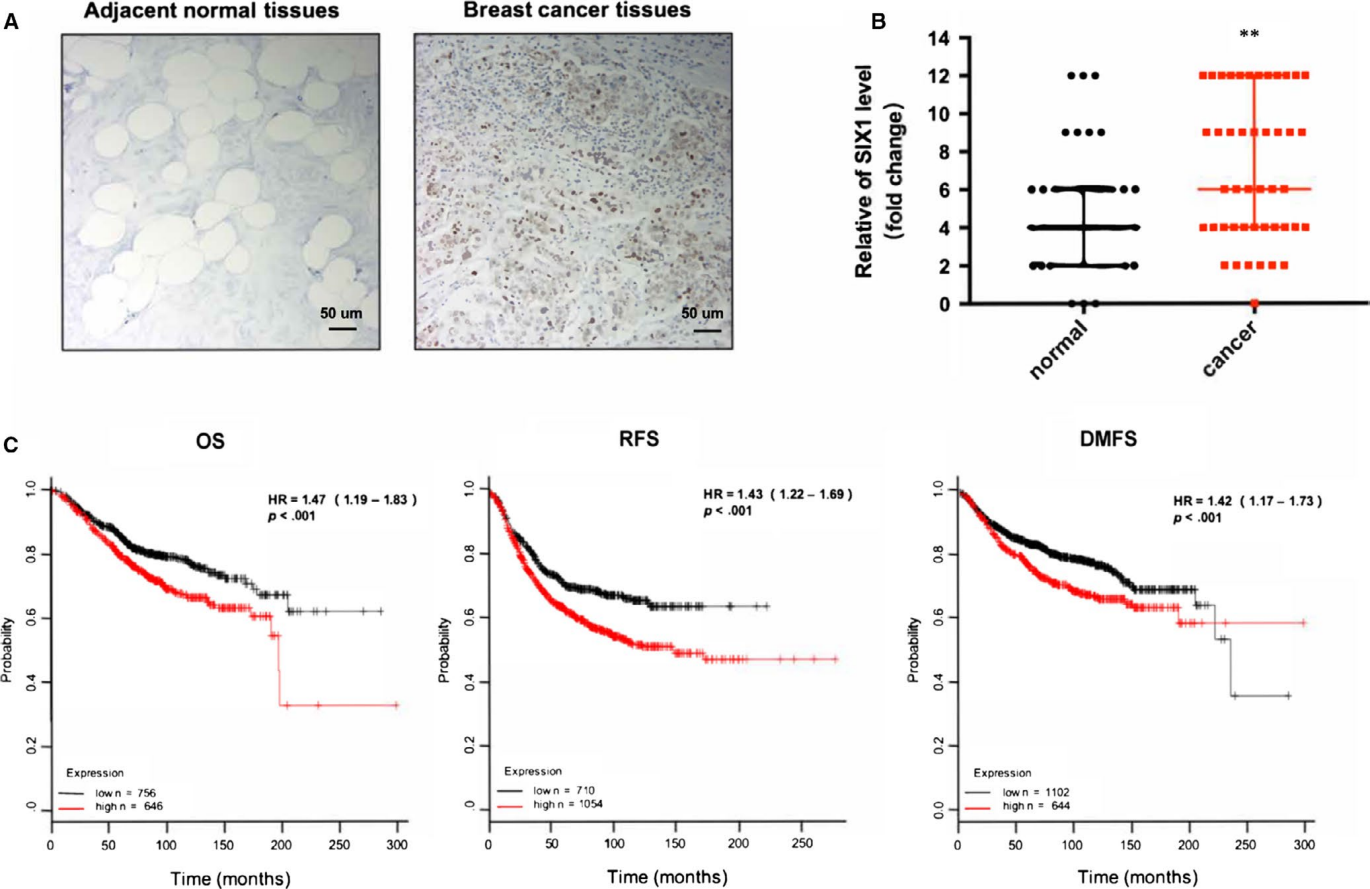
SIX‐1 was up‐regulated in BC. A, Representative images of immunohistochemical (IHC) staining of SIX‐1 staining in 45 BC tissues and 45 adjacent normal tissues. B, Chi‐square analysis of the SIX‐1 level in 45 BC tissues and 45 adjacent normal tissues. **represents Kruskal‐Wallis test *P* < .01. C, Kaplan‐Meier curve depicting the overall survival (OS) of 1402 BC patients, the relapse‐free survival(RFS) of 1754 BC patients and the distant metastasis‐free survival (DMFS) of 1746 GC patients. The data were analysed by a Kaplan‐Meier plotter (http://kmplot.com/analysis/). The programme was allowed to automatically determine the best cut‐off between expression groups, and samples were censored at the ‘all’ threshold

To clarify whether progression of BC correlated with dysregulation of SIX‐1, we first detected the expression of SIX‐1 on a tissue chip by immunohistochemistry (IHC), which contains 45 pairs of BC tissues and paired non‐tumour breast tissues. As determined by IHC staining, SIX‐1 expression was significantly up‐regulated in BC tissues compared with paired non‐tumour breast tissues (Figure 1A,B). Importantly, up‐regulation of SIX‐1 showed strong association with lymph node metastasis and AJCC stage in BC patients (Table 1). These data suggested that high expression of SIX‐1 in BC may contribute to BC progression. To further draw attention to whether increased expression of SIX‐1 affects survival probability of BC patient, we screened a public database (http://kmplot.com/analysis/index.php?p=service%26cancer=breast) and confirmed that the overall survival (OS), recurrence‐free survival (RFS) and distant metastasis‐free survival (DMFS) of BC patients were longer in the low‐SIX‐1‐expression group than in the high‐SIX‐1‐expression group (Figure 1C), suggesting that SIX‐1 plays a pivotal role in BC development.

**Table 1:** Association of SIX‐1 level with clinicopathological parameters of patients with breast cancer

Characteristics	Total	SIX‐1		*P*
		Low	High	
Age (y)				
≤60	36	14	22	.76
>60	9	4	5	
pStage				
I/II	20	7	13	.54
III/IV	25	11	14	
Tumor size				
＜4 cm	31	13	18	.69
≥4 cm	14	5	9	
T				
T 1/2	41	18	23	.09
T 3	4	0	4	
N				
N 0/1	31	16	15	.02
N 2/3	14	2	12	
Number of positive lymph nodes				
＜5	32	16	16	.03
≥5	13	2	11	
AJCC stage				
1A‐2B	31	16	15	.02
3A‐3C	14	2	12	

### 3.2 SIX‐1 promoted invasion and migration of BC cells in vitro and facilitated metastases in vivo

**Figure 2:**
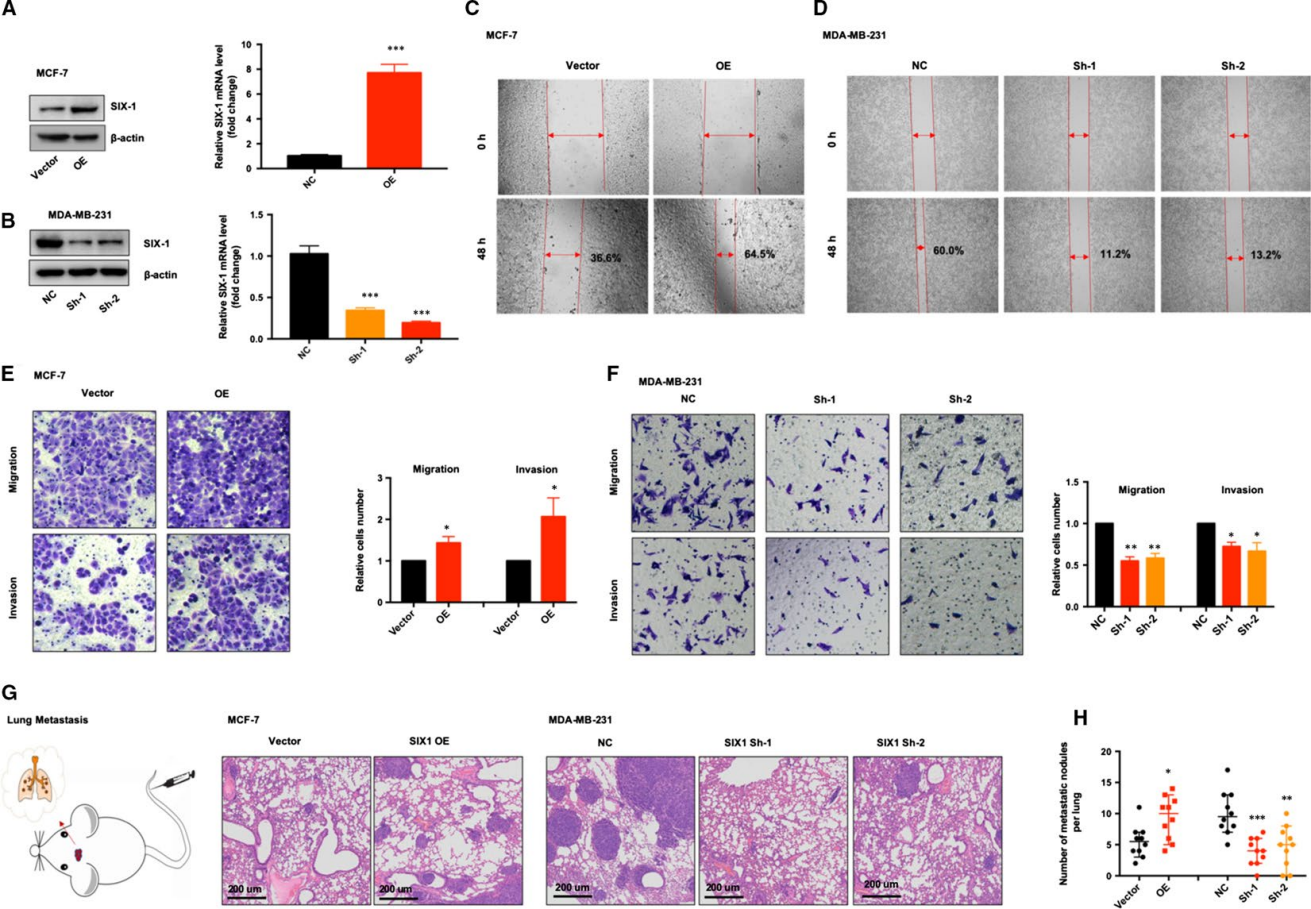
SIX‐1 promoted BC cell invasion and migration. A, Figure 2: MCF‐7 cells were transfected with lentiviral vectors encoding SIX‐1 (OE) or control vector (vector). The expression of SIX‐1 was detected by Western blotting and qRT‐PCR. The values shown are expressed as the means ± SEM. ***represents Student's *t* test *P* < .001. B, MDA‐MB‐231 cells were transfected with lentiviral vectors encoding SIX‐1 short hairpin RNA vector (Sh‐1 or Sh‐2) or control vector (NC). The expression of SIX‐1 was detected by Western blotting and qRT‐PCR. The values shown are expressed as the means ± SEM. ***represents Student's *t* test *P* < .001. C and D, Wound healing assay showed overexpression of indicated MCF (C) or MDA‐MB‐231 (D) cell clones. Representative images are shown. The number represents the proportion of healed areas. E and F, Transwell migration and invasion assay of indicated MCF (E) or MDA‐MB‐231 (F) cell clones. Representative images of the experiments are shown. *represents Student's *t* test *P < .05 and **P < .01. G, Indicated cells were injected into nude mice (n = 10 for each group) via the tail vein and animals were sacrificed at 8 wk after the injections. Representative HE staining of lung tissue samples is shown. H, The number of lung metastatic foci observed in each group. *represents Kruskal‐Wallis test *P* < .05, **P < .01 and ***P < .001

To verify the pro‐metastatic role of SIX‐1 in BC, we overexpressed SIX‐1 via lentivirus infection in MCF‐7 (MCF‐7‐SIX‐1‐OE), a BC cell line with low metastatic potential and knockdown SIX‐1 via lentivirus infection in MDA‐MB‐231 (MDA‐MB‐231‐SIX‐1‐Sh), a BC cell line with high metastatic potential,^27^ for further investigation (Figure 2A,B). Wound healing was significantly (*P* value < .05) increased in cells ectopically expressing SIX‐1, with 64.5% area healed in SIX‐1 expression set compared to 36.6% of empty vector, in MCF‐7 cell line. In MDA‐MB‐231 cell line, SIX‐1 knockdown led to 11.2% (Sh‐1) and 13.2% (Sh‐2) area being healed, compared to 60.0% of empty vector set (*P* value < .05) (Figure 2C,D). Consistent with the results of wound healing assay, transwell migration and invasion assay further confirmed that overexpression of SIX‐1 gained stronger invasion and migration ability, while knockdown of SIX‐1 blocked the invasion and migration ability in BC cells (Figure 2E,F). Moreover, in vivo mice model of lung metastases further showed that overexpression of SIX‐1 triggered cell metastases, as evidenced by more metastatic nodules, was found in mice injected with MCF‐7‐SIX‐1‐OE cells through vena caudalis, while knockdown of SIX‐1 could reverse the pro‐metastatic effect of MDA‐MB‐231 cells (Figure 2G,H). Collectively, both in vitro and in vivo functional studies demonstrated that SIX‐1 possessed pro‐metastatic ability in BC progression.

### 3.3 SIX‐1 promoted BC progression by regulating EMT

**Figure 3:**
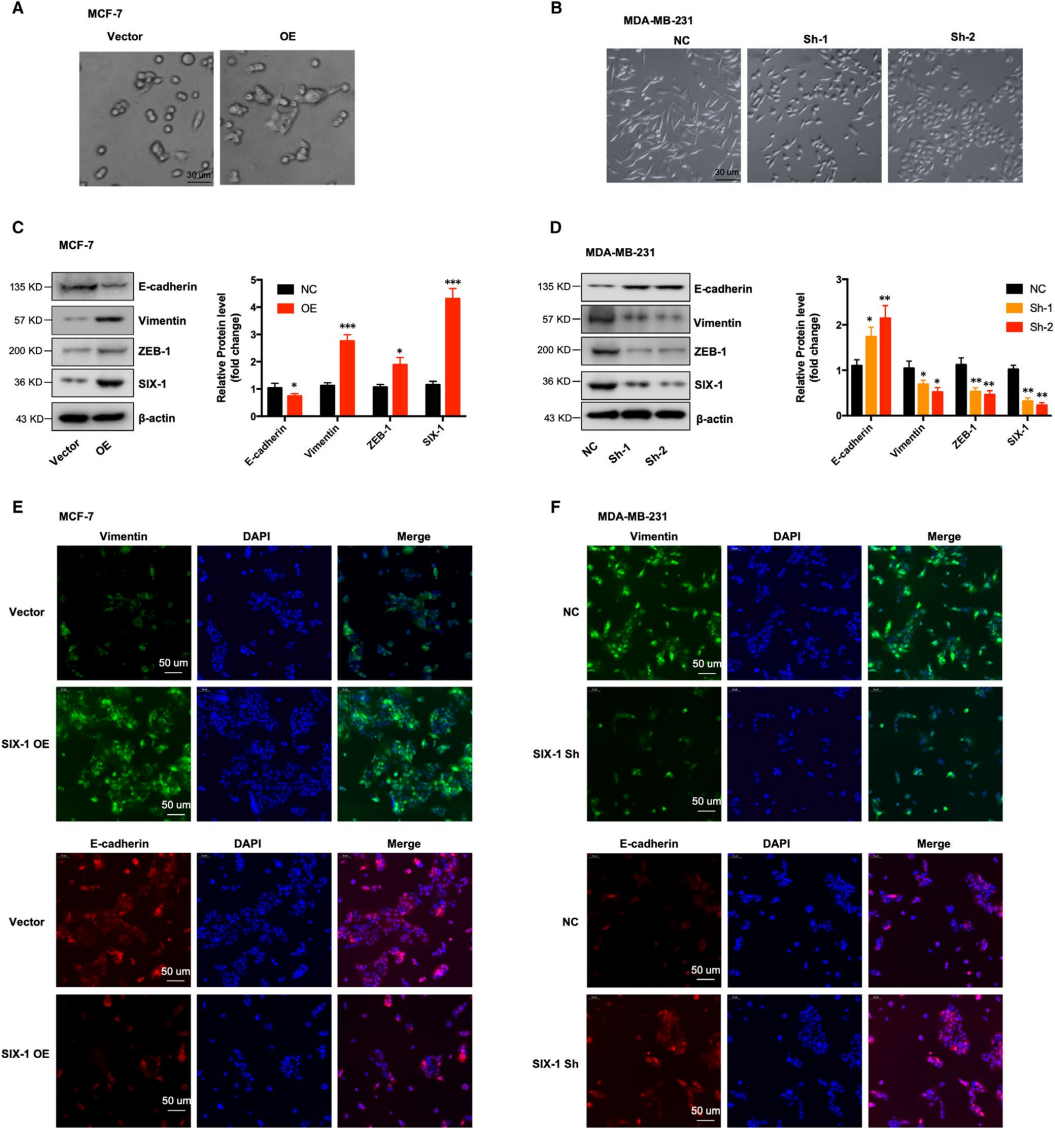
SIX‐1 promoted BC progression by regulating EMT. A and B, Phase‐contrast micrographs of indicated MCF‐7 (A) or MDA‐MB‐231 (B) cell clones. C and D, Western blotting reveal the change of epithelial markers and mesenchymal markers in of indicated MCF (C) or MDA‐MB‐231 (D) cell clones. The mean level of the indicated protein from three independent biological replicates is shown on the histogram. The values shown are expressed as the means ± SEM. *represents Student's *t* test *P < .05, **P < .01 and ***P < .001. E and F, Immunofluorescence staining reveal the change of epithelial markers and mesenchymal markers in of indicated MCF (E) or MDA‐MB‐231 (F) cell clones

Previous studies showed that SIX‐1 usually exerts its pro‐metastatic ability by regulating EMT.^13^, ^28^ Morphological assay showed that overexpression of SIX‐1 lead to disorganization of the epithelial morphology of MCF7 cells, leading to a fibroblast‐like morphology transition, whereas down‐regulation of SIX‐1 leads to reverse process (Figure 3A,B), indicating that SIX‐1 may contribute to EMT in BC. Consistent with the morphological results, Western blot assay and IF staining illustrated that overexpression of SIX‐1 caused a significant decrease in the expression of epithelial marker E‐cadherin, while lead to increase expression of mesenchymal markers, such as vimentin (Figure 3C,E). However, down‐regulation of SIX‐1 leads to up‐regulation of E‐cadherin, and down‐regulation of vimentin and ZEB1 (Figure 3D,F). These data taken together confirmed that SIX‐1 contributes to BC metastases at least partly by inducing EMT.

### 3.4 SIX‐1 induced EMT through controlling TGF‐β and ZEB1 expression

**Figure 4:**
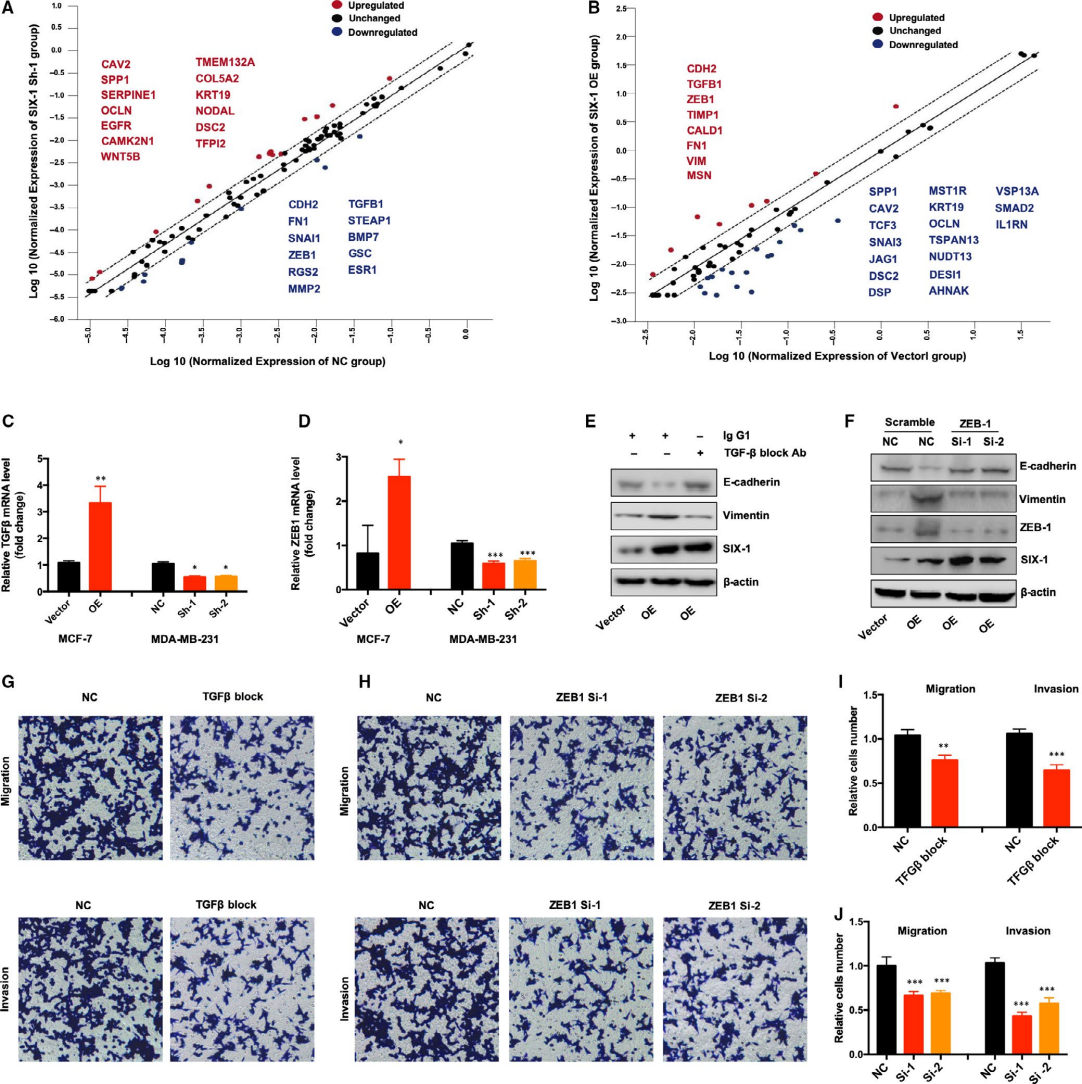
SIX‐1 induced EMT through controlling TGF‐β and ZEB1 expression. A and B, The scatter plot compares the normalized expression of every gene on the array between the SIX‐1 Sh‐1 group and NC group in MDA‐MB‐231 cells (A) or SIX‐1 OE group and vector group in MCF‐7 cells (B) by plotting them against one another to quickly visualize changes in gene expression. The central line indicates unchanged gene expression. The dotted lines indicate the 2‐fold regulation threshold. Data points beyond the dotted lines in the upper left and lower right sections meet the 2‐fold regulation threshold. C, The expression of TGF‐β indicated cell lines were detected by qRT‐PCR. * represents Student's *t* test *P < .05 and **P < .01. D, The expression of TGF‐β indicated cell lines were detected by qRT‐PCR. * represents Student's *t* test *P < .05 and ***P < .001. E, MCF‐7 SIX‐1 OE cells (OE) or control cells (vector) were treated with anti‐TGF‐β blocking monoclonal antibody or the corresponding IgG_1_. The expression of Indicated protein was detected by Western blotting. F, MCF‐7 SIX‐1 OE cells (OE) or control cells (vector) were transfected with ZEB1 siRNA or scramble control. The expression of Indicated protein was detected by Western blotting. G and I, Transwell migration and invasion assay of MCF‐7 SIX‐1 OE cells with anti‐TGF‐β blocking monoclonal antibody or the corresponding IgG_1_ treatment. Representative images of the experiments are shown (G). ** represents Student's *t* test **P < .01 and ***P < .001 (I). H and J, Transwell migration and invasion assay of MCF‐7 SIX‐1 OE cells with ZEB1 siRNA or scramble control transfection. Representative images of the experiments are shown (H). *** represents Student's *t* test *P* < .001 (J)

To explain in‐depth the mechanisms of SIX‐1‐induced EMT in BC metastases, we performed PCR array assay in the SIX‐1 Sh‐1 group and NC group in MDA‐MB‐231 cells or SIX‐1 OE group and vector group in MCF‐7 cells. In the SIX‐1 down‐regulation group, 13 genes were up‐regulated more than twice, such as OCLN, CAV2, KRT19 and DSC2, and 8 genes were down‐regulated, such as TGFB1, ZEB1, FN1 and CDH2 (Figure 4A). Meanwhile, in the SIX‐1 up‐regulation group, the 8 genes were up‐regulated more than twice, such as FN1, VIM, TGFB1 and ZEB1, and 17 genes were down‐regulated more than twice, such as VSP13A, DESI1, CAV2 and TCF3 (Figure 4B). It was known that TGF‐β1 and ZEB1 can regulate the EMT process of various cancer cells.^29^, ^30^ The changes of these two genes in BC cells were consistent with SIX‐1, so we selected TGFB1 and ZEB1 for further study. PCR analysis also verified that both TGF‐β1 and ZEB1 levels were significantly increased when the MCF7 cells overexpression of SIX‐1, whereas both TGF‐β1 and ZEB1 levels were significantly decreased when SIX‐1 was knocked down in MDA‐MB‐231 cells (Figure 4C,D), indicating that SIX‐1‐induced EMT in BC metastases may depend on TGF‐β1 and ZEB1. In addition, Western blot assay showed that inhibition of TGF‐β1 by using TGF‐β blocking antibodies or suppression of ZEB1 by using siRNA in MCF‐7‐SIX‐1‐OE cells could significantly reverse SIX‐1‐induced EMT (Figure 4E,F). Similarly, transwell assay also revealed that inhibition of TGF‐β1 (Figure 4G,I) or ZEB1 (Figure 4H,J) in MCF‐7‐SIX‐1‐OE cells could reverse SIX‐1‐induced cell invasion and migration. These data demonstrated that TGF‐β1 and ZEB1 are responsible for SIX‐1‐induced EMT in BC metastases.

### 3.5 The increased expression of ZEB1 induced by SIX‐1 is partially dependent on miR‐200 family

**Figure 5:**
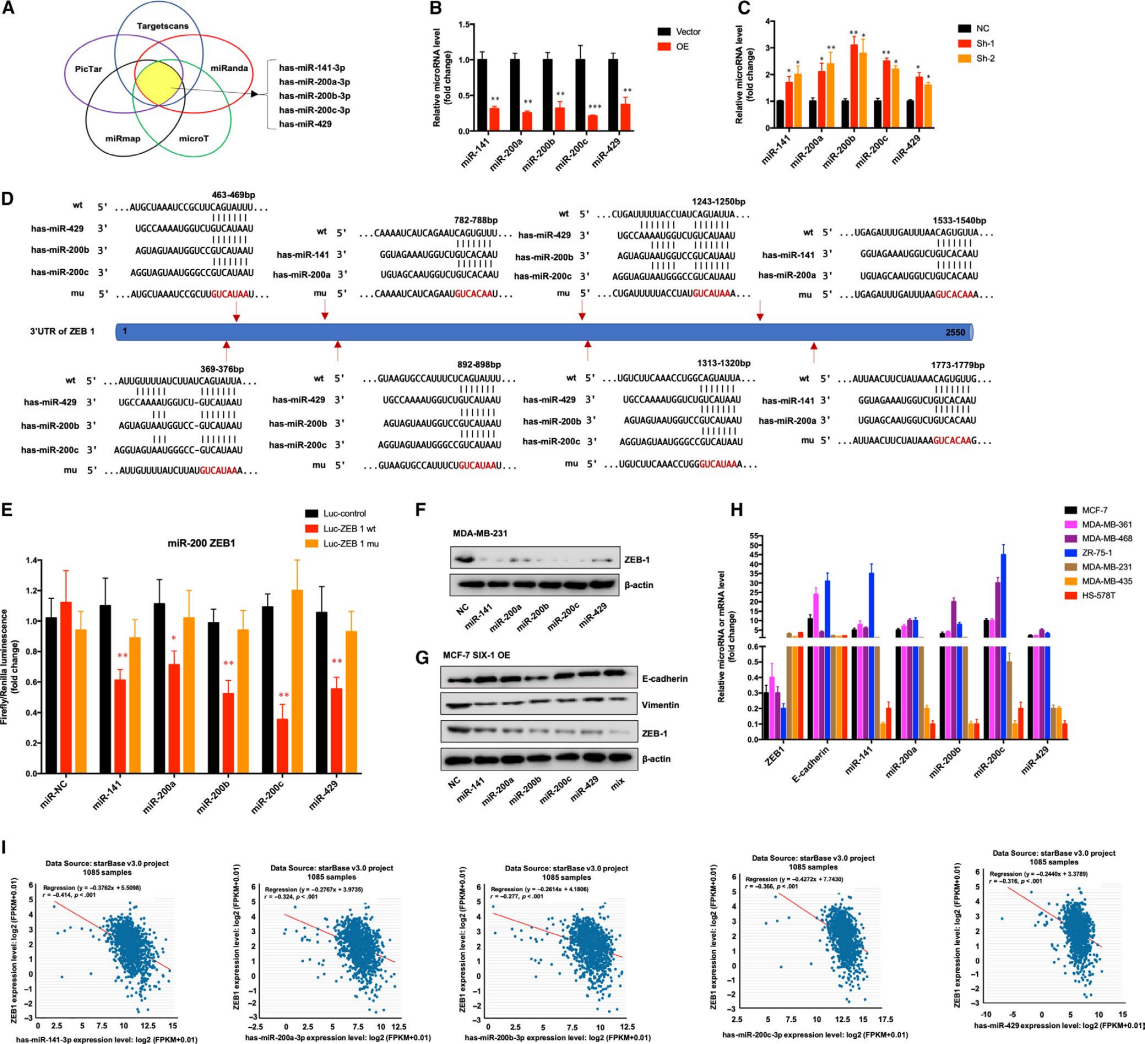
MiR‐200 family is necessary for SIX‐1‐induced ZEB1 signalling in BC. A, Venn diagram displaying miRNAs computationally predicted to target ZEB1 from PicTar, TargetScanS, miRanda, microT and miRmap database. B, The expression of miR‐200 family in MCF‐7 SIX‐1 OE cells or indicated control cells were detected by qRT‐PCR. ** represents Student's *t* test **P < .01 and ***P < .001. C, The expression of miR‐200 family in MDA‐MB‐231 SIX‐1 Sh cells or indicated control cells were detected by qRT‐PCR. * represents Student's *t* test *P < .05, **P < .01 and ***P < .001. D, Schematic of the predicted miR‐200 family binding sites in the ZEB1 3’UTR and indicated mutant binding sites. E, Luciferase activity assay for targeting the 3′‐UTR of ZEB1 by miR‐200 family. The wild‐type and mutant miR‐200s target sequences of ZEB1 were fused to the luciferase reporter and were transfected into the control vector (Luc‐ZEB1 wt and Luc‐ZEB1 mu). Luc‐ZEB1 wt, Luc‐ZEB1 mu or the control vector was cotransfected with miR‐200s or a miRNA negative control into HEK293T cells, and the luciferase activity was measured. Data are presented as the relative ratio of firefly luciferase activity to Renilla luciferase activity. * represents Student's *t* test *P < .05 and **P < .01. F, The expression of ZEB1 was determined by Western blotting in MDA‐MB‐231 cells transfected with a miR‐200s mimic or negative control. β‐Actin was used as the loading control. G, The expression of indicated protein was determined by Western blotting in MCF‐7 SIX‐1 OE cells transfected with a miR‐200s mimic or negative control. β‐Actin was used as the loading control. H, miR‐200s and E‐cadherin, ZEB1 mRNAs levels were measured by real‐time PCR in breast cancer cell lines. I, The correlation between SIX‐1 mRNA level and the miR‐200s transcript level was measured in 1085 BC tissues using starBase v3.0 project (http://starbase.sysu.edu.cn)

MiRNAs, as post‐transcriptional regulator, have been recently found to play an important role in tumour progression, and we hypothesized that post‐transcriptional regulation by miRNAs may represent an upstream regulatory mechanism of ZEB1 expression. We firstly used several web‐based target prediction algorithms (TargetScanS, miRanda, pictar, miRmap and microT) to identify miRNAs that could potentially target ZEB 1. MiR‐141, miR‐200a‐3p, miR‐200b‐3p, miR‐200c‐3p and miR‐429, which all belong to the miR‐200 family, were simultaneously identified by several algorithms as potential regulators of ZEB1 expression (Figure 5A). Consistently, PCR assay further confirmed that the expression of all miR‐200 family members, especially miR‐200c, was dramatically decreased in MCF‐7 cells when SIX‐1 was overexpressed (Figure 5B). However, a significant increase in expression of miR‐200 family members could be found in MDA‐MB‐231‐SIX‐1‐KD cells (Figure 5C). Bioinformatics analysis revealed that there were multiple binding sites of miR‐200 family on 3’‐untranslated region (UTR) of ZEB1 mRNA (Figure 5D). Then, we also found the luciferase activity of the reporter constructs was most significantly reduced when miR‐200 family mimic constructs, including miR‐141, miR‐200a‐3p, miR‐200b‐3p, miR‐200c‐3p and miR‐429, were cotransfected with the wild‐type ZEB1 3’‐UTR reporter (Luc‐ZEB1‐wt), respectively, into MCF‐7 cells, while there were no significant changes when miR‐200 family mimic constructs were cotransfected with ZEB1 3’‐UTR reporter in which the binding sites of miR‐200 family were mutated (Luc‐ZEB1‐mu) (Figure 5E). Meanwhile, Western blotting assay also confirmed the protein level of ZEB1 was significantly reduced after miR‐200 family mimic transfection into MDA‐MB‐231 cells (Figure 5F). In addition, Western blotting results revealed that transfected miR‐200 family mimics, respectively, or mixed in MCF‐7‐SIX‐1‐OE cells could reverse SIX‐1‐mediated EMT (Figure 5G), suggesting that miR‐200 family may be necessary for SIX‐1‐induced ZEB1 signalling in BC metastases. To further demonstrate that miR‐200 family‐induced ZEB1 in EMT is a common phenomenon of BC, we texted the expression of ZEB1, E‐cadherin, and miR‐200 family members in multiple BC cell lines and found that miR‐200 family showed a global influence on ZEB1‐mediated EMT of BC cells (Figure 5H). Then, the correlation between SIX‐1 mRNA level and the miR‐200s transcript level was measured in 1085 BC tissues using starBase v3.0 project (http://starbase.sysu.edu.cn). Consistently, the results further showed a strong negative correlation between miR‐200 family and ZEB1 in 1085 BC samples (Figure 5I), confirming that the increased expression of ZEB1 induced by SIX‐1 is partially dependent on miR‐200 family.

### 3.6 lncRNA ATB was up‐regulated by TGF‐β1 and was associated with the miR‐200 family

**Figure 6:**
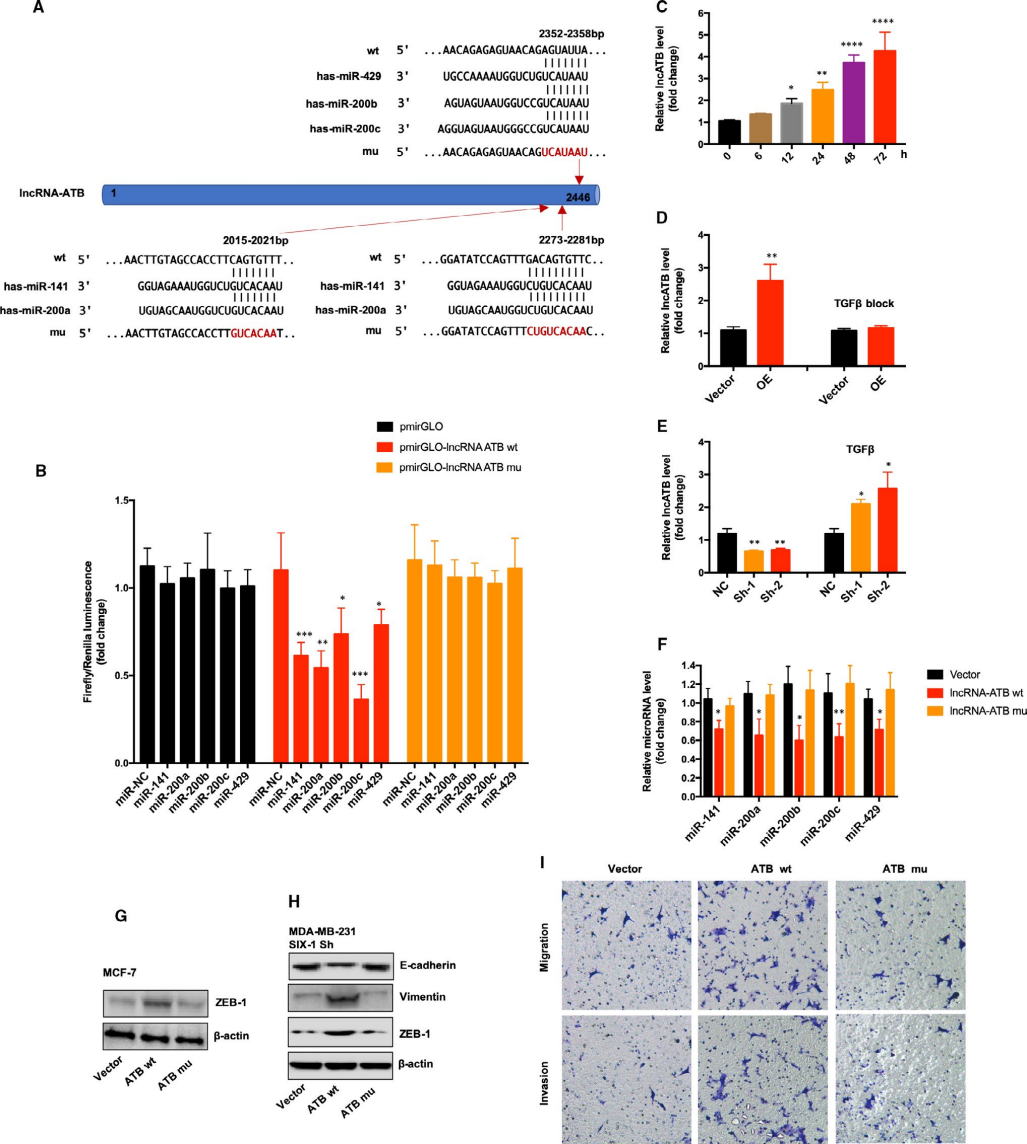
lncRNA ATB was necessary for SIX‐1‐miR‐200s axis. A, Schematic of the predicted miR‐200 family binding sites on lncRNA ATB transcript and indicated mutant binding sites. B, The Luciferase activity in HEK293T cells cotransfected with miR‐200s mimics and luciferase reporters containing nothing (pmirGLO), lncRNA ATB (pmirGLO‐lncRNA ATB wt) or mutant transcript (pmirGLO‐lncRNA ATB mu). Data are presented as the relative ratio of firefly luciferase activity to renilla luciferase activity. * represents Student's *t* test *P < .05, **P < .01 and ***P < .001. C, Relative expression of lncRNA ATB in MCF‐7 cells treated with TGF‐β1 for the indicated time was measured by qRT‐PCR. D, Relative expression of lncRNA ATB in MCF‐7 SIX‐1 OE cells or vector cells with or without TGF‐β blocking monoclonal antibody treatment was measured by qRT‐PCR. E, Relative expression of lncRNA ATB in MDA‐MB‐231 SIX‐1 Sh cells or indicated NC cells with or without TGF‐β treatment was measured by qRT‐PCR. F, Relative expression of miR‐200s in MCF‐7 cells with indicated plasmid transfection was measured by qRT‐PCR. G, The expression of ZEB1 in MCF‐7 cells with indicated plasmid transfection was measured by Western blotting. H, The expression of ZEB1, E‐cadherin and vimentin in MDA‐MB‐231 SIX‐1 Sh1 cells with indicated plasmid transfection was measured by Western blotting. β‐Actin was used as the loading control. I, Transwell migration and invasion assay of MDA‐MB‐231 SIX‐1 Sh1 cells with indicated plasmid transfection. Representative images of the experiments are shown

We next evaluated how SIX‐1 controlled miR‐200 family in BC metastases. Previous study showed that the lncRNA‐activated by TGF‐β (lncRNA ATB), which was up‐regulated by TGF‐β1, could promote EMT by competitively binding the miR‐200 family.^30^ Our data showed that TGF‐β1 level increased significantly as SIX‐1 expression level up‐regulated in BC cells. Therefore, we hypothesis that lncRNA ATB may be able to function as a ceRNA for miR‐200 family in SIX‐1‐induced EMT of BC. To verify our hypothesis, we found that multiple miR‐200 family binding sites existed on LncATB (Figure 6A). For further confirmation, we constructed luciferase reporters containing the 3’ 1000nt of lncRNA ATB, which contains wild‐type (pmirGLO‐lncRNA ATB wt), or the binding sites of miR‐200 family were mutated (pmirGLO‐lncRNA ATB mu). We found that the luciferase activity of the reporter constructs was most significantly reduced when miR‐200 family mimic constructs, including miR‐141, miR‐200a‐3p, miR‐200b‐3p, miR‐200c‐3p and miR‐429, were cotransfected with the wild‐type IncATB 3′‐UTR reporter into MCF‐7 cells but not empty vector or mutant reporter vector, indicating a strong interaction between lncRNA ATB and miR‐200 family (Figure 6B). To confirm that lncRNA ATB could up‐regulated by TGF‐β1 in BC cells, we treated MCF‐7 with TGF‐β1 and found a robust increase of lncRNA ATB expression during time course (Figure 6C). Moreover, lncRNA ATB expression was dramatically increased when the cells overexpressed SIX‐1, while the level of lncRNA ATB was decreased when the MCF‐7‐SIX‐1‐OE cells blocked by TGF‐β1 blocking antibodies (Figure 6D). Consistently, compared with corresponding control cells, MDA‐MB‐231‐SIX‐1‐KD cells showed a decreased expression of lncRNA ATB. However, the expression level of lncRNA ATB was increased when MDA‐MB‐231‐SIX‐1‐KD cells was treated with TGF‐β1 (Figure 6E), illustrating that SIX‐1 could regulation the expression level of lncRNA ATB via TGF‐β1. In addition, transfection with wild‐type IncATB constructs in MDA‐MB‐231‐SIX‐1‐KD cells could effectively reverse the increase of miR‐200 family expression, while transfected with IncATB which the binding sites of miR‐200 family were mutated (lncRNA ATB mu) showed no significant change (Figure 6F), suggesting that lncRNA ATB is required for SIX‐1‐mediated miR‐200s expression. In addition, transfection with wt‐lncRNA ATB could also lead to change of ZEB1 expression but mutant lncRNA ATB not (Figure 6G), indicating that ZEB1 could also be regulated by lncRNA ATB. Importantly, Western blot assay further confirmed that transfection with wt‐lncRNA ATB but not mutant lncRNA ATB in MDA‐MB‐231‐SIX‐1‐KD cells could reverse the EMT process (Figure 6H). Transwell assay also showed that the invasion and migration ability of MDA‐MB‐231‐SIX‐1‐KD cells was reversed when transfected with wt‐lncRNA ATB but not mutant lncRNA ATB (Figure 6I). Altogether, our result confirmed that lncRNA ATB may be able to function as a ceRNA for miR‐200 in SIX‐1‐induced EMT of BC.

### 3.7 The clinicopathological characteristics associated with SIX‐1, lncATB, miR‐200c and ZEB1 expression in BC patients

**Figure 7:**
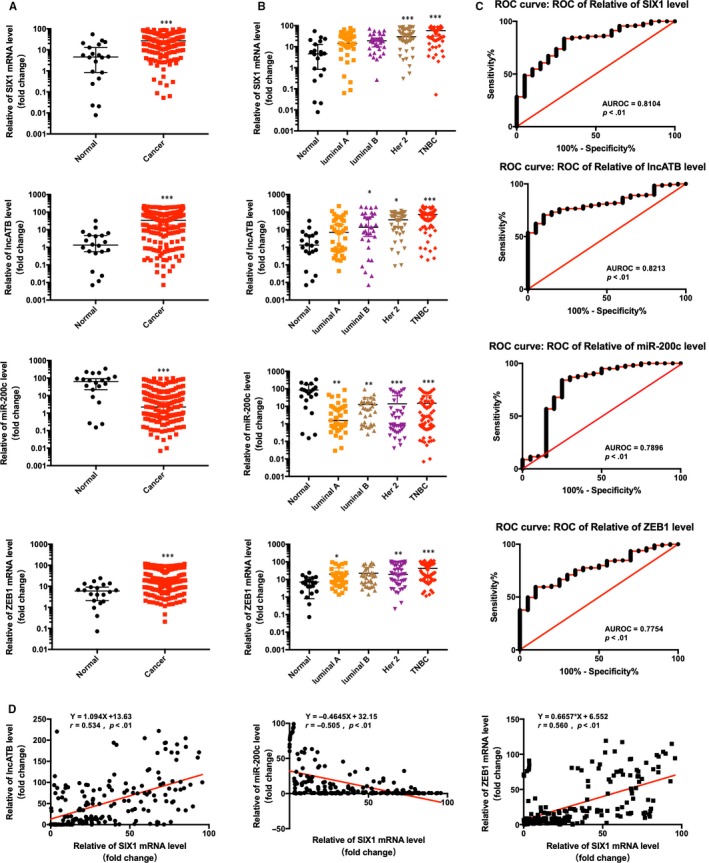
The clinicopathological characteristics associated with SIX‐1, lncATB, miR‐200c and ZEB1 expression in BC patients. A, The relative expression of SIX‐1, lncATB, miR‐200c‐3p and ZEB1 in normal breast and breast cancer tissues. *** represents Student's *t* test ***P < .001. B, The relative expression of SIX‐1, lncATB, miR‐200c‐3p and ZEB1 in breast cancer tissues with different molecular subtypes. * represents Student's *t* test *P < .05, **P < .01 and ***P < .001. C, Diagnostic efficacy of SIX‐1, lncATB, miR‐200c‐3p or ZEB1 in the breast cancer patients was assessed by area under the receiver operating characteristic (AUROC) curves. D, The correlation between SIX‐1 mRNA level and the lncATB transcript level (left), the miR‐200c‐3p transcript level (middle) or ZEB1 mRNA level (right) was measured in 183 BC tissues

To further define the role of SIX‐1‐LncATB‐miR‐200‐ZEB1 axis in human breast cancer patients and the correlation between SIX‐1, lncATB, miR‐200c‐3p and ZEB1, we measured the relative expression levels of SIX‐1, lncATB, miR‐200c‐3p and ZEB1 in 203 samples, including 20 normal breast tissues and 183 breast cancer tissues. We found that compared with normal breast tissue, the expression of SIX‐1, lncATB and ZEB1 was increased in breast cancer tissue, while the expression of miR‐200c‐3p was decreased (Figure 7A). Then, we analysed the expression of these four molecules in breast cancer tissues with different molecular subtypes, including 39 luminal A cases, 32 luminal B cases, 44 Her2‐positive cases and 68 triple‐negative cases. The results showed that SIX‐1, lncATB and ZEB1 expression levels revealed that higher expression level was observed in Her2‐positive and triple‐negative breast cancer and lower level in normal mammary glands (Figure 7B). Meanwhile, the expression level of miR‐200c‐3p was lower in Her2‐positive and triple‐negative breast and higher in normal mammary glands (Figure 7B). To further evaluate the diagnostic efficacy of SIX‐1‐LncATB‐miR‐200‐ZEB1 axis in BC, the area under the receiver operating characteristic (AUROC) curve was calculated. As shown in Figure 7C, all four molecules, SIX‐1 (AUC = 0.8104, *P* < .01), lncATB (AUC = 0.8213, *P* < .01), miR‐200c‐3p (AUC = 0.7896, *P* < .01) and ZEB1 (AUC = 0.7754, *P* < .01), have certain diagnostic capabilities for breast cancer. Moreover, we also found that SIX‐1 mRNA levels were significantly positively correlated with lncATB transcript level (*r* = .534, *P* < .01) and negatively correlated with miR‐200c‐3p level (*r* = −.505, *P* < .01), while SIX‐1 mRNA levels were significantly positively correlated with the ZEB1 mRNA levels (*r* = .560, *P* < .01) (Figure 7D).

## 4 DISCUSSION

This study we found that SIX‐1 expression was frequently up‐regulated in BC patients, and high level of SIX‐1 was associated with lymph node metastasis and correlated with unfavourable OS, RFS and DMFS. Mechanistically insight, we found that SIX‐1 up‐regulated the expression of lncATB, which exerts critical pro‐metastatic role in BC by directly binding to the miR‐200 family, especially for miR‐200c, to induce EMT and promote metastases. Thus, SIX‐1 could act as an important diagnostic marker as well as significant therapeutic targets for BC patients.

Numerous conclusive evidence had indicated that overexpression of SIX‐1 in advanced malignancies is a key regulator for tumour metastases.^10^, ^31^, ^32^, ^33^ Previous study showed indications that SIX‐1 promoted BC metastases by regulating TGF‐β1‐induced EMT, which was consistent with our results (Figures 1 and 2). By using EMT‐related PCR array assay in MCF‐7‐SIX‐1‐OE and MDA‐MB‐231‐SIX‐1‐KD cells, we further found and confirmed that besides TGF‐β1, ZEB1 are also responsible for SIX‐1‐induced EMT in BC metastases (Figure 3). ZEB1 is an important transcription factor activated during EMT, and ZEB1 activation was associated with poor overall survival in advanced BC.^34^ However, there was a complex relationship between SIX‐1, TGF‐β1, ZEB1 and EMT, and the potential regulators involved in this process remain largely unclear. Indeed, SIX‐1 could directly up‐regulate ZEB1 expression. There are two tandem homeodomain consensus binding sites present in ZEB1 promoter DNA, and these homeobox sites were highly conserved among several species including Mus musculus, Homo sapiens, Bos primigenius and Danio rerio.^35^ However, we found that when SIX‐1 was overexpressed in breast cancer cells, the multiple of ZEB1 mRNA increase was significantly lower than that of ZEB1 protein increase. This phenomenon suggests that in addition to direct transcription regulation, there are other ways for SIX‐1 to regulate the expression of ZEB1. In our study, we found that SIX‐1 can not only directly promote the transcription of ZEB1, but also indirectly promote the expression of ZEB1 by inhibiting the expression of miR‐200 family, further clarifying the mechanism of SIX‐1 promoting the elevation of ZEB1 protein. Among which, miR‐200 family, especially miR‐200c, has been reported to be crucial for ZEB1 activation.^21^, ^36^ Using starBase analysis, we showed a strong negative correlation between miR‐200 family and ZEB1 in 1085 BC samples (Figure 5I). Besides, miR‐200 family showed a global influence on ZEB1‐mediated EMT of BC cells (Figure 5H). In addition, transfected miR‐200 family mimics in MCF‐7‐SIX‐1‐OE cells could reverse SIX‐1‐mediated EMT (Figure 5G). Thus, our data provided strong evidences suggesting that miR‐200 family is necessary for SIX‐1‐induced ZEB1 signalling in BC metastases. However, no explanations are currently available on either how SIX‐1 controlled miR‐200 family in BC metastases or how TGF‐β1 involved in these complex processes. Therefore, we further investigated and payed attention to the lncRNA‐activated by TGF‐β (lncRNA ATB), which was up‐regulated by TGF‐β1, has been reported could promote EMT by competitively binding the miR‐200 family.^30^ Luciferase reporter assay showed a strong interaction between lncRNA ATB and miR‐200 family, as evidenced by the activity of the reporter constructs was most significantly reduced when miR‐200 family mimic constructs were cotransfected with the wild‐type IncATB 3’‐UTR reporter into MCF‐7 cells (Figure 6B). Moreover, our results also confirmed that SIX‐1 could regulate the expression level of lncRNA ATB via TGF‐β1 and that lncRNA ATB is required for SIX‐1‐mediated miR‐200s expression, suggesting that SIX‐1/lncRNA ATB/miR‐200s axis is one of the possible mechanisms that control BC metastases. Meanwhile, we also found that transfection with wt‐lncRNA ATB could also lead to change of ZEB1 expression but not mutant lncRNA ATB (Figure 6G), indicating that ZEB1 could also be regulated by lncRNA ATB. Taken together, we confirmed that lncRNA ATB may be able to function as a ceRNA for miR‐200s in SIX‐1 induced EMT of BC. We finally emphasized the clinical significance of SIX‐1 in BC metastases by assessing the correlation between the SIX‐1 expression level and the clinicopathological characteristics, and showed that SIX‐1 was a promising diagnosis marker to predict poorly clinical outcomes of advanced BC.

**Figure 8:**
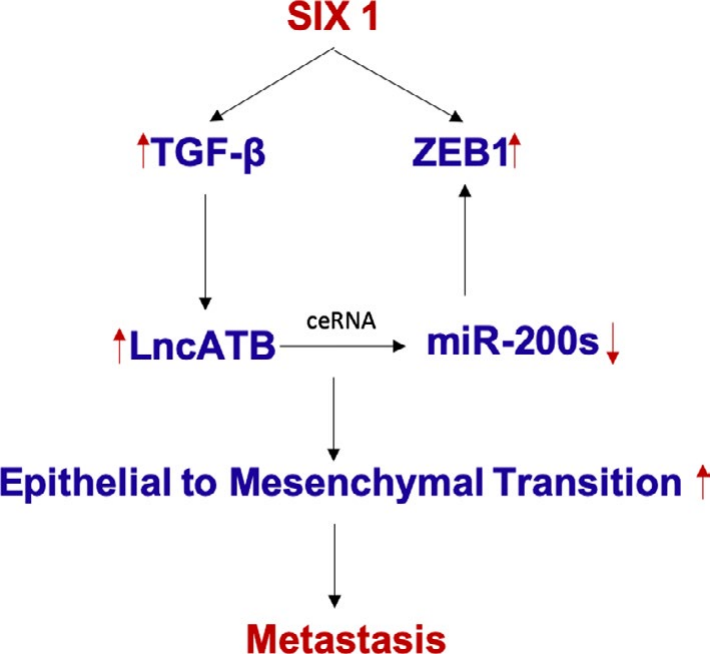
Schematic model depicting that SIX‐1 exerts its pro‐metastatic role in BC through lncATB/miR‐200s axis of EMT signalling pathway

In conclusion, SIX‐1 exerts its pro‐metastatic role in BC through lncATB/miR‐200s axis of EMT signalling pathway and could act as an important diagnostic marker as well as a significant therapeutic target for clinically advanced BC (Figure 8).

## CONFLICT OF INTEREST

The authors declare no conflict of interest in this study.

## AUTHORS’ CONTRIBUTIONS

BW and KW designed the study; LZ, SJ and SY collected the data; SJ, SY, SP and PX were involved in contribution of new reagents or analytical tools; LZ, HC and XL analysed the data; LZ, SJ, XL, KW and BW prepared the manuscript.

## ETHICS APPROVAL AND CONSENT TO PARTICIPATE

A total of 183 of BC tissues and 20 non‐tumour breast tissues were randomly obtained with informed consent from surgery patients of BC (First Affiliated Hospital of Xi'an Jiaotong University, Xi'an, Shaanxi, China). Patients enrolled for this study were not received any chemotherapy, radiotherapy or targeted therapy. The tissues collected were snap‐frozen in liquid nitrogen immediately for further RNA and protein extraction. All the human studies were performed according to ethical consent granted from the Clinical Research Ethics Committee of the Xi'an Jiaotong University.

Male nude mice (4‐week‐old, n = 10/group) were purchased from Beijing HFK Biotechnology Co, Ltd. Animal study was approved by the Ethics Committee of the Xi'an Jiaotong University.

## Data Availability

The data that support the findings of this study are available from the corresponding author upon reasonable request.

## References

[1] Ward EM , DeSantis CE , Lin CC , et al. Cancer statistics: Breast cancer in situ. CA Cancer J Clin. 2015;65:481‐495. 26431342 10.3322/caac.21321

[2] DeSantis C , Ma J , Bryan L , Jemal A . Breast cancer statistics, 2013. CA Cancer J Clin. 2014;64(1):52‐62. 24114568 10.3322/caac.21203

[3] Hill BS , Sarnella A , D'Avino G , Zannetti A . Recruitment of stromal cells into tumour microenvironment promote the metastatic spread of breast cancer. Semin Cancer Biol. 2019;60:202‐213. 31377307 10.1016/j.semcancer.2019.07.028

[4] Scimeca M , Urbano N , Bonfiglio R , et al. Novel insights into breast cancer progression and metastasis: A multidisciplinary opportunity to transition from biology to clinical oncology. Biochim Biophys Acta Rev Cancer. 2019;1872:138‐148. 31348975 10.1016/j.bbcan.2019.07.002

[5] Thiery JP , Acloque H , Huang RY , Nieto MA . Epithelial‐mesenchymal transitions in development and disease. Cell. 2009;139:871‐890. 19945376 10.1016/j.cell.2009.11.007

[6] Mego M , Mani SA , Cristofanilli M . Molecular mechanisms of metastasis in breast cancer–clinical applications. Nat Rev Clin Oncol. 2010;7:693‐701. 20956980 10.1038/nrclinonc.2010.171

[7] Thiery JP . Epithelial‐mesenchymal transitions in tumour progression. Nat Rev Cancer. 2002;2:442‐454. 12189386 10.1038/nrc822

[8] Radisky DC . Defining a role for the homeoprotein Six1 in EMT and mammary tumorigenesis. J Clin Invest. 2009;119:2528‐2531. 19726879 10.1172/JCI40555 PMC2735897

[9] Ford HL , Kabingu EN , Bump EA , Mutter GL , Pardee AB . Abrogation of the G2 cell cycle checkpoint associated with overexpression of HSIX1: a possible mechanism of breast carcinogenesis. Proc Natl Acad Sci USA. 1998;95:12608‐12613. 9770533 10.1073/pnas.95.21.12608 PMC22878

[10] Reichenberger KJ , Coletta RD , Schulte AP , Varella‐Garcia M , Ford HL . Gene amplification is a mechanism of Six1 overexpression in breast cancer. Cancer Res. 2005;65:2668‐2675. 15805264 10.1158/0008-5472.CAN-04-4286

[11] Coletta RD , Christensen KL , Micalizzi DS , Jedlicka P , Varella‐Garcia M , Ford HL . Six1 overexpression in mammary cells induces genomic instability and is sufficient for malignant transformation. Cancer Res. 2008;68:2204‐2213. 18381426 10.1158/0008-5472.CAN-07-3141

[12] McCoy EL , Iwanaga R , Jedlicka P , et al. Six1 expands the mouse mammary epithelial stem/progenitor cell pool and induces mammary tumors that undergo epithelial‐mesenchymal transition. J Clin Invest. 2009;119:2663‐2677. 19726883 10.1172/JCI37691 PMC2735909

[13] Micalizzi DS , Christensen KL , Jedlicka P , et al. The Six1 homeoprotein induces human mammary carcinoma cells to undergo epithelial‐mesenchymal transition and metastasis in mice through increasing TGF‐beta signaling. J Clin Invest. 2009;119:2678‐2690. 19726885 10.1172/JCI37815 PMC2735914

[14] Di Leva G , Garofalo M , Croce CM . MicroRNAs in cancer. Annu Rev Pathol. 2014;9:287‐314. 24079833 10.1146/annurev-pathol-012513-104715 PMC4009396

[15] Farazi TA , Hoell JI , Morozov P , Tuschl T . MicroRNAs in human cancer. Adv Exp Med Biol. 2013;774:1‐20. 23377965 10.1007/978-94-007-5590-1_1 PMC3704221

[16] Lages E , Ipas H , Guttin A , Nesr H , Berger F , Issartel JP . MicroRNAs: molecular features and role in cancer. Front Biosci. 2012;17:2508‐2540. 10.2741/4068 PMC3815439 22652795

[17] Korpal M , Kang Y . The emerging role of miR‐200 family of microRNAs in epithelial‐mesenchymal transition and cancer metastasis. RNA Biol. 2008;5:115‐119. 19182522 10.4161/rna.5.3.6558 PMC3532896

[18] Feng X , Wang Z , Fillmore R , Xi Y . MiR‐200, a new star miRNA in human cancer. Cancer Lett. 2014;344:166‐173. 24262661 10.1016/j.canlet.2013.11.004 PMC3946634

[19] Bracken CP , Gregory PA , Kolesnikoff N , et al. A double‐negative feedback loop between ZEB1‐SIP1 and the microRNA‐200 family regulates epithelial‐mesenchymal transition. Cancer Res. 2008;68:7846‐7854. 18829540 10.1158/0008-5472.CAN-08-1942

[20] Burk U , Schubert J , Wellner U , et al. A reciprocal repression between ZEB1 and members of the miR‐200 family promotes EMT and invasion in cancer cells. EMBO Rep. 2008;9:582‐589. 18483486 10.1038/embor.2008.74 PMC2396950

[21] Brabletz S , Brabletz T . The ZEB/miR‐200 feedback loop–a motor of cellular plasticity in development and cancer? EMBO Rep. 2010;11:670‐677. 20706219 10.1038/embor.2010.117 PMC2933868

[22] Humphries B , Wang Z , Li Y , Jhan JR , Jiang Y , Yang C . ARHGAP18 Downregulation by miR‐200b Suppresses Metastasis of Triple‐Negative Breast Cancer by Enhancing Activation of RhoA. Cancer Res. 2017;77:4051‐4064. 28619708 10.1158/0008-5472.CAN-16-3141

[23] Pang Y , Liu J , Li X , et al. MYC and DNMT3A‐mediated DNA methylation represses microRNA‐200b in triple negative breast cancer. J Cell Mol Med. 2018;22:6262‐6274. 30324719 10.1111/jcmm.13916 PMC6237581

[24] Li C . New functions of long noncoding RNAs during EMT and tumor progression. Cancer Res. 2019;79:3536‐3538. 31308135 10.1158/0008-5472.CAN-19-1205

[25] Wang Z , Zhao Y , Smith E , et al. Reversal and prevention of arsenic‐induced human bronchial epithelial cell malignant transformation by microRNA‐200b. Toxicol Sci. 2011;121:110‐122. 21292642 10.1093/toxsci/kfr029 PMC3080188

[26] Zhou L , Shang Y , Liu C , et al. Overexpression of PrPc, combined with MGr1‐Ag/37LRP, is predictive of poor prognosis in gastric cancer. Int J Cancer. 2014;135:2329‐2337. 24706505 10.1002/ijc.28883 PMC4277329

[27] Nigro A , Mauro L , Giordano F , et al. Recombinant arabidopsis HSP70 sustains cell survival and metastatic potential of breast cancer cells. Mol Cancer Ther. 2016;15:1063‐1073. 26939699 10.1158/1535-7163.MCT-15-0830

[28] Ono H , Imoto I , Kozaki K , et al. SIX1 promotes epithelial‐mesenchymal transition in colorectal cancer through ZEB1 activation. Oncogene. 2012;31:4923‐4934. 22286765 10.1038/onc.2011.646

[29] Gregory PA , Bert AG , Paterson EL , et al. The miR‐200 family and miR‐205 regulate epithelial to mesenchymal transition by targeting ZEB1 and SIP1. Nat Cell Biol. 2008;10:593‐601. 18376396 10.1038/ncb1722

[30] Yuan JH , Yang F , Wang F , et al. A long noncoding RNA activated by TGF‐beta promotes the invasion‐metastasis cascade in hepatocellular carcinoma. Cancer Cell. 2014;25:666‐681. 24768205 10.1016/j.ccr.2014.03.010

[31] Behbakht K , Qamar L , Aldridge CS , et al. Six1 overexpression in ovarian carcinoma causes resistance to TRAIL‐mediated apoptosis and is associated with poor survival. Cancer Res. 2007;67:3036‐3042. 17409410 10.1158/0008-5472.CAN-06-3755

[32] Yu Y , Khan J , Khanna C , Helman L , Meltzer PS , Merlino G . Expression profiling identifies the cytoskeletal organizer ezrin and the developmental homeoprotein Six‐1 as key metastatic regulators. Nature Med. 2004;10:175‐181. 14704789 10.1038/nm966

[33] Ng KT , Man K , Sun CK , et al. Clinicopathological significance of homeoprotein Six1 in hepatocellular carcinoma. Br J Cancer. 2006;95:1050‐1055. 17008870 10.1038/sj.bjc.6603399 PMC2360701

[34] Li RH , Chen M , Liu J , et al. Long noncoding RNA ATB promotes the epithelial−mesenchymal transition by upregulating the miR‐200c/Twist1 axe and predicts poor prognosis in breast cancer. Cell Death Dis. 2018;9:1171. 30518916 10.1038/s41419-018-1210-9 PMC6281614

[35] Cieply B , Farris J , Denvir J , Ford HL , Frisch SM . Epithelial‐mesenchymal transition and tumor suppression are controlled by a reciprocal feedback loop between ZEB1 and Grainyhead‐like‐2. Cancer Res. 2013;73:6299‐6309. 23943797 10.1158/0008-5472.CAN-12-4082 PMC3806457

[36] Brabletz S , Bajdak K , Meidhof S , et al. The ZEB1/miR‐200 feedback loop controls Notch signalling in cancer cells. EMBO J. 2011;30:770‐782. 21224848 10.1038/emboj.2010.349 PMC3041948

